# A prospective clinical study of Primo-Lacto: A closed system for colostrum collection

**DOI:** 10.1371/journal.pone.0206854

**Published:** 2018-11-12

**Authors:** Alexandria I. Kristensen-Cabrera, Jules P. Sherman, Henry C. Lee

**Affiliations:** 1 Department of Pediatrics, Division of Neonatology, Stanford University School of Medicine, Palo Alto, CA, United States of America; 2 Hasso Plattner Institute of Design, Stanford University, Palo Alto, CA, United States of America; University of Illinois, UNITED STATES

## Abstract

**Background:**

Colostrum is the first nutritional liquid that comes out of the breast during lactation. Colostrum collection can be challenging due to the small volume produced, and because breast pumps are not designed for colostrum collection. Besides pumping colostrum, the generally accepted practice is to use any available container to hand-express colostrum. Transfer between containers may lead to contamination, higher chance of infection and loss of colostrum. Our aim was to understand if a dedicated colostrum collection system (Primo-Lacto, Maternal Life, LLC, Palo Alto, CA) is more effective than standard hospital practice.

**Methods:**

Mothers who delivered preterm infants < 34 weeks gestation and mothers with non-latching infants were approached within 24 hours of delivery. Surveys were distributed to participating patients (n = 67), and nurses or lactation consultants (n = 89). Mothers compared ease of use, their confidence level and satisfaction with the amount collected during standard practice vs. the colostrum collection system. Nurses or lactation consultants compared ease of use, differences in colostrum loss and time invested collecting. Quantitative data were analyzed using the Wilcoxon signed rank test and qualitative data were analyzed with grounded theory methods.

**Results:**

For mothers, ease of use and confidence were significantly better when they used the colostrum collection system than when they used the standard collection procedure, and this difference was true for both hand and pump expression (p<0.01). Nurses and lactation consultants perceived that ease of use was better, and percent of colostrum lost was significantly less with the colostrum collection system for both hand and pump expression. The collection times were not significantly different between the colostrum collection system and standard practice.

**Conclusion:**

The colostrum collection system is a tool to help facilitate successful colostrum collection and improve the experience both for clinicians and patients.

## Introduction

Recent research has shown that the combination of breastmilk and infant saliva may enhance innate immunity [1]. However, most preterm infants are not able to feed orally [2, 3]. Similar to breastfeeding in term infants, studies have indicated that oral colostrum priming improves immune protection in preterm infants, enhances the oral immunomicrobial environment, and reduces clinical sepsis [4–6].

Colostrum has a high concentration of nutrients and antibodies. For example, it contains high concentrations of secretory immunoglobulin (IgA) which is an antibody that serve as a first line of immune defense from pathogenic organisms [7]. In addition, colostrum contains high concentrations of leukocytes which protect the neonate from harmful bacteria and viruses. Typically, only 2–10 milliliters of colostrum are expressed per feed. Quantities may be even less for a mother with a preterm infant and therefore it is important to capture and deliver every drop to the neonate [8].

Premature infants fed exclusively with human colostrum and human milk tend to vomit less than premature infants fed with formula. Colostrum improves long term health outcomes such as the prevention of obesity, cardiovascular disease, and diabetes. Moreover, human colostrum may have a significant role protecting against chronic diseases and allergies [9].

A positive experience during the colostral phase may encourage women to continue breastfeeding long-term, but complications are extremely common. Maternal feeding attitudes, education and the support women receives from her partner and/or family during the first four weeks of breastfeeding greatly affect whether or not she continues breastfeeding for six months to one year [10]. Women should receive appropriate guidance and education while in the hospital in order to help identify and manage common breastfeeding issues [10].

About 10% of births in the United States are preterm and 17% of mothers in the United States do not initiate breastfeeding due to issues such as birth complications, lack of support, or personal and cultural preferences [11].

Published research has shown that the best practice for expressing colostrum is “Hands-On Pumping” (HOP). HOP is the combination of hand expression and pump expression [5]. This combination increases colostrum production with a higher caloric value [12]. However, there is a gap in research regarding the optimal tool set for collecting and storing colostrum.

Introducing hand expression to a mother unable to breastfeed after birth increases her chances of breastfeeding long term [3]. Based on clinical focus groups at 6 different US hospitals, colostrum is hand-expressed into plastic spoons, tiny plastic vials or medicine cups every few hours. Each session only produces a few milliliters of colostrum. After expressing into any manner of work-around containers, a feeding syringe is used to vacuum up the colostrum and feed it to the newborn. When using a pump, the colostrum often gets stuck in the pump’s valve and/or sides of the attached bottles. The lactation consultant or nurse has to then scrape the colostrum out of the pump valve or bottle with a spoon or swab. This collection and transfer practice is awkward, causes significant colostrum loss and may increase the chance of contamination and infection to the neonate. Moreover, difficulties expressing colostrum the first few days may present a significant psychological barrier to new mothers [13].

Current practice relies on “work-arounds” rather than a dedicated system to improve a mother’s chances of successfully expressing her colostrum.

A colostrum collection system (Primo-Lacto, Maternal Life, LLC, Palo Alto, CA) is intended for use when (1) mothers cannot or choose not to nurse their newborn or (2) the newborn cannot nurse due to trouble latching, limited sucking reflex, or prematurity.

Over five years, this colostrum collection system traversed 19 design iterations, clinical focus groups at six different California hospitals, a clinical study at three different hospital sites in the United States and Food and Drug Administration (FDA) registration before it was finally manufactured and distributed for sale in 2017.

**Fig 1:**
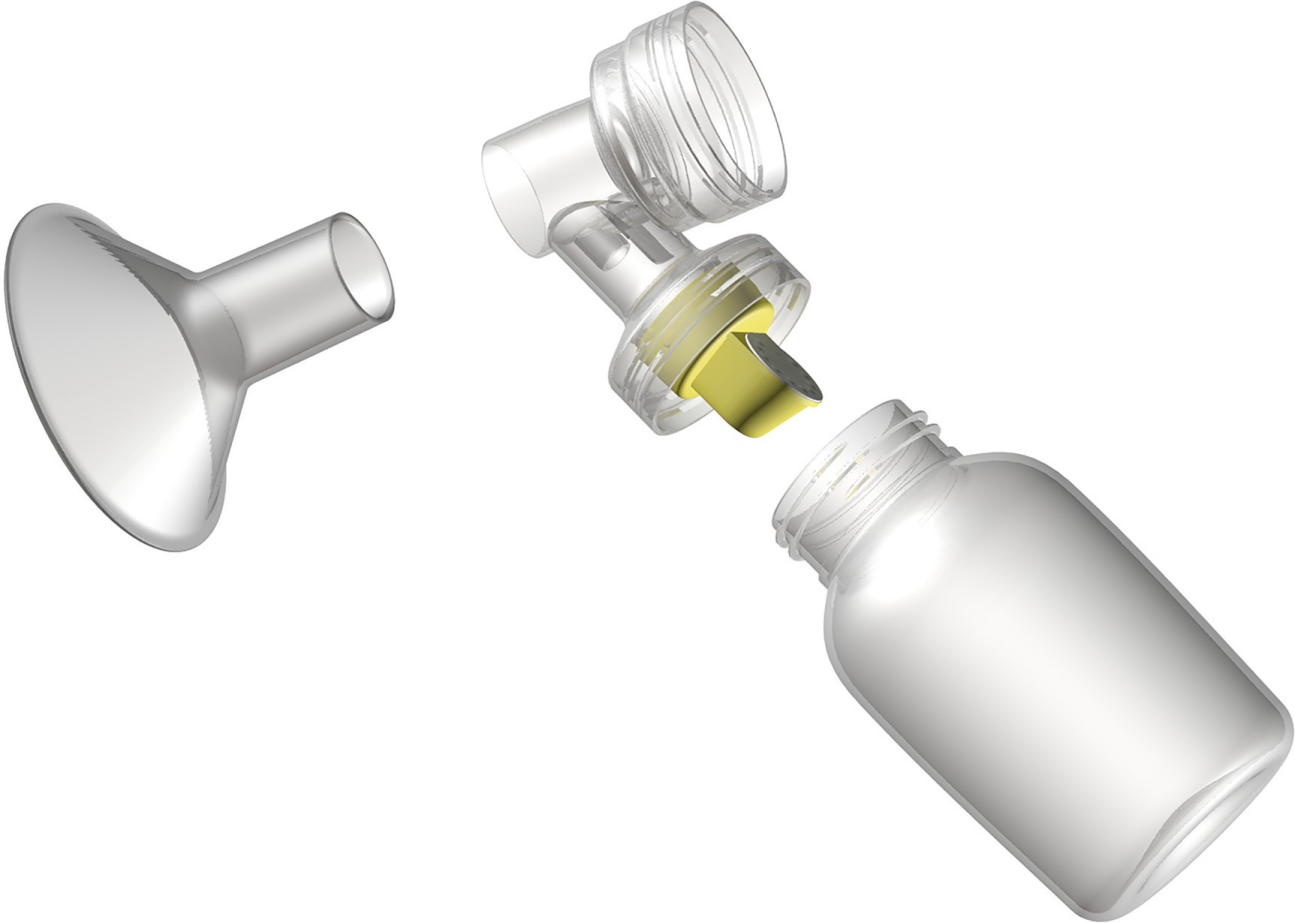
A standard hospital pump assembly.

**Fig 2:**
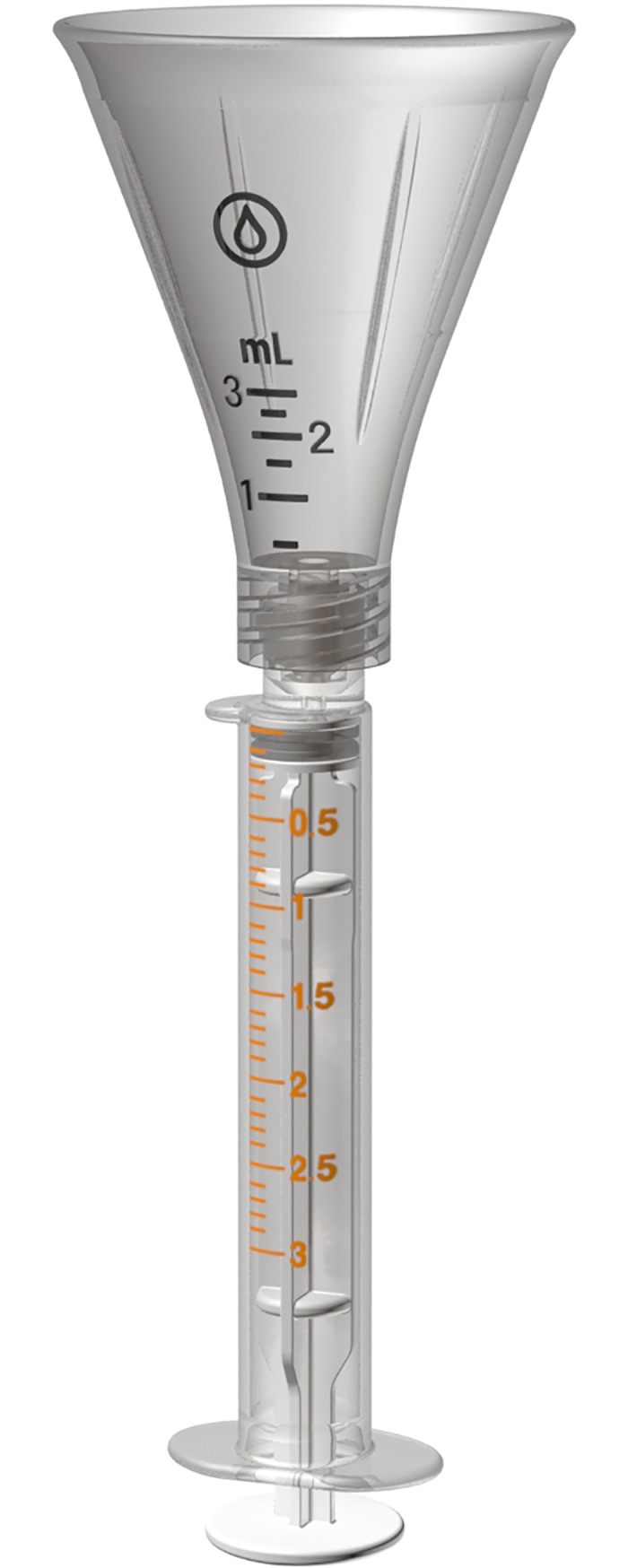
A standard hospital pump assembly with the colostrum collection system adapter and syringe connected.

**Fig 3:**
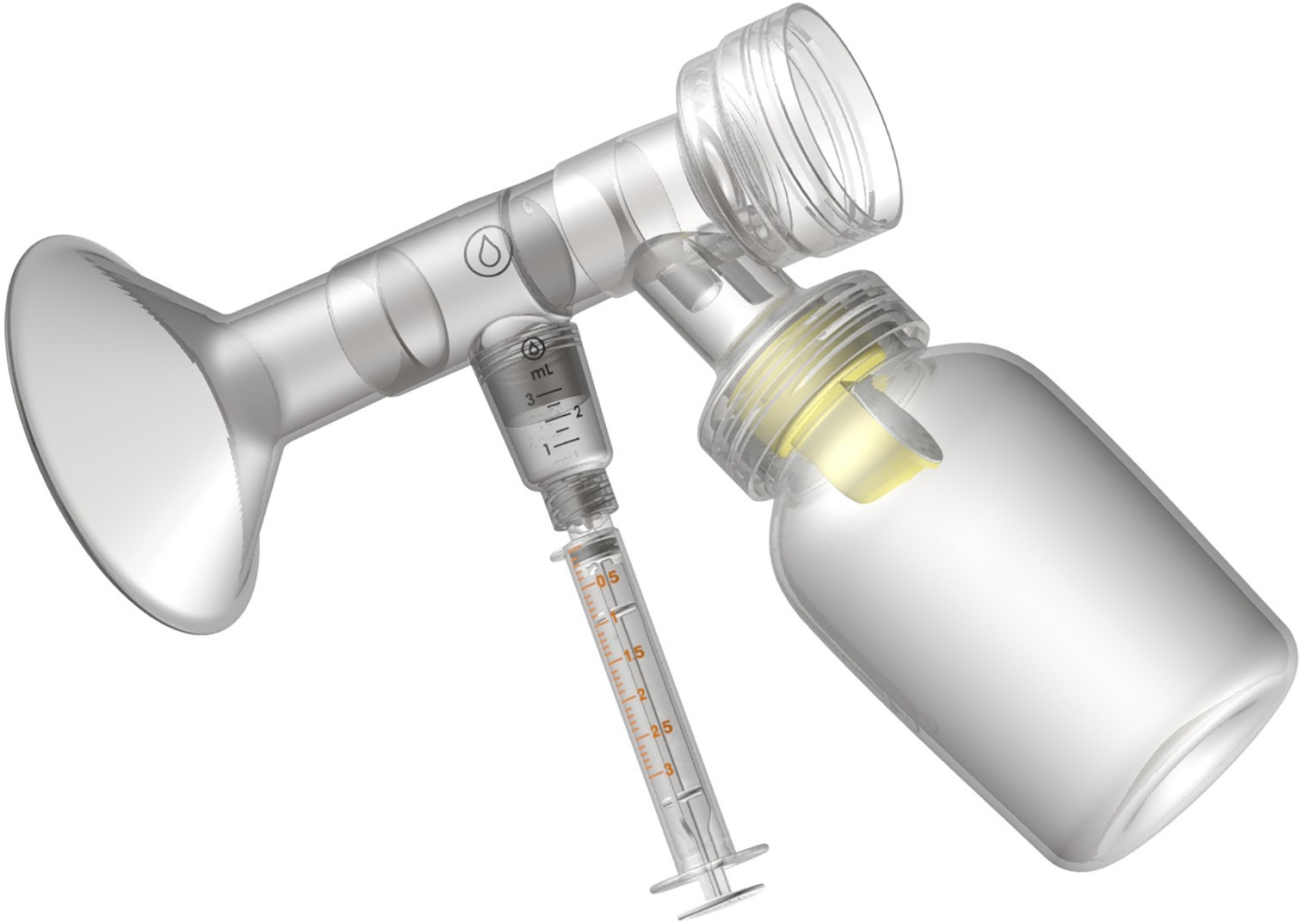
The colostrum collection system hand expression funnel shown connected to a syringe.

The colostrum collection system is a two-part closed system kit comprised of two breast pump adapters (for double pumping) and a hand expression funnel. Both accessories connect directly to an enteral syringe so that colostrum is routed directly into the syringe. The colostrum collection system can interchangeably connect to the most commonly used feeding syringes (ENFit and slip tip) and hospital grade pump system (Fig 1). The colostrum collection system encourages hands-on pumping by incorporating both a pump adapter (Fig 2) and a hand-expression funnel (Fig 3).

The aim of this study was for mothers and providers to compare the colostrum collection system with standard practice (i.e. standard pump and hand expression). The significance of this study lies in the desire to understand how a human-centered product design may help or hinder colostrum collection for mothers who cannot breastfeed after birth.

## Materials and methods

This study was approved by the Institutional Review Boards at Sharp Mary Birch Hospital in San Diego, California, John Muir Medical Center in Walnut Creek, California and Memorial Hospital of South Bend, Indiana; and all clinical investigation was conducted according to the principles expressed in the Declaration of Helsinki. Informed, written consent was obtained from all participants.

In this paper we define breastfeeding as feeding a baby colostrum or mature breast milk directly from the breast. The design of the study was prospective, with each mother serving as her own control. Through testing the colostrum collection system at three different hospital sites, we assessed the patient’s experience and nurse experience and workflow. No data were recorded on mothers who were screened but did not participate in the study. All survey responses were de-identified. Logs were kept on a secure hospital-owned password protected and encrypted computer. This study was approved by the Institutional Review Boards at Sharp Mary Birch Hospital in San Diego, California, John Muir Medical Center in Walnut Creek, California and Memorial Hospital of South Bend, Indiana. Written consent was obtained from all participants.

The setting was the mother’s hospital room for the first three days after birth. Participating hospitals included Sharp Mary Birch Hospital in San Diego, California, John Muir Medical Center in Walnut Creek, California and Memorial Hospital of South Bend, Indiana. Data were collected from March 2016-January 2017.

Mothers who delivered preterm infants < 34 weeks gestation were approached within 24 hours after delivery, when they typically would be expected to have started pumping their breasts (assuming that they planned on providing human milk to their infant). We focused on infants <34 weeks gestation because studies have shown that preterm infants benefit greatly from receiving their mothers colostrum. In addition, mothers of non-latching infants or infants with other health conditions that prevent breastfeeding were approached. Assuming that a mother had already attempted to express colostrum, they were invited to participate in this study. Women who were already past the colostral phase were excluded from the study. At all three testing hospitals the Medela Symphony Pump was used both for standard pump practice and with the Primo-Lacto adapter.

### Data Collection

The intervention occurred, and data were collected within three days of birth (i.e., the colostral phase). Data were collected by mothers when they first tested the equipment and at the end of their colostral phase. Nurses and lactation consultants judged the equipment and colostrum loss throughout each patient’s process, however waited to record their perceptions until the end of testing each patient.

The sequence of the four lactation procedures was the same for all mothers: mothers started alternating practices from the beginning of the colostral period (immediately after birth) until the end of the third day. The study used scaled data from participant’s (mothers and nurses or lactation consultants) responses to questions comparing both the Primo-Lacto colostrum collection system and standard methods. Standard methods mean standard hospital practice including the typical assistance from a nurse or lactation consultant. Standard practice in all three testing hospitals is pumping colostrum in a bottle or container and hand expressing in some sort of cup. By allowing participants to write comments under each scale question, we were able to uncover deeper insights.

Maternal Life supplied hospital staff with the following directions.

### Protocol for nurse or lactation consultant

Suggest alternate method with each session: alternate between standard practice and the intervention. Standard practice pumping and hand expression, and then the colostrum collection system methods (repeat) Record:

- EGA—estimated gestational age (at birth)
- Birth weight
- Gravida, Para
  1. Have mother hand express using standard method (5 min).
  2. Have mother pump express (5 min.) using the standard method.
  3. Have mother pump express using the colostrum collection system (5 min.); select the appropriate sized flange for mothers. Attach the colostrum collection system adapter in between the flange and valve part of the pump. Attach the reservoir to the adapter. Attach a 3 ml NeoMed slip enteral syringe to the nipple on the adapter by press fitting it into the hole. Attach a bottle to the base of the pump valve part. Begin pumping session. Once the mother has finished, pull plunger on the syringe down to vacuum the colostrum from the adapter’s reservoir. Pull syringe off of adapter, cap, label and bring to the Neonatal Intensive Care Unit (NICU) or infant for feeding. (Note: Adapter/reservoir can be hand washed with soap and water between uses.)
  4. Have mother hand express using the colostrum collection system (5 min); attach a NeoMed 3ml slip enteral syringe to the funnel by press fitting it into the hole. Make sure to keep the plunger up. When mother has finished a hand expression session, pull down on the syringe plunger to vacuum the colostrum from the funnel into the syringe. Cap, label and bring to NICU, or infant for feeding. (Note: Funnel can be hand washed with soap and water between uses.) The colostrum collection system adapter can be hand washed with soap and water, autoclaved or steam cleaned between uses.

Possible Risks:

There are no health risks. The pump adapter device does not come into contact with the mother during use. The hand-expression funnel is simply held in the mother’s hand while she expresses. The colostrum collection system is made from hydrophobic biocompatible plastic similar to standard breast pump accessories.

**Fig 4:**
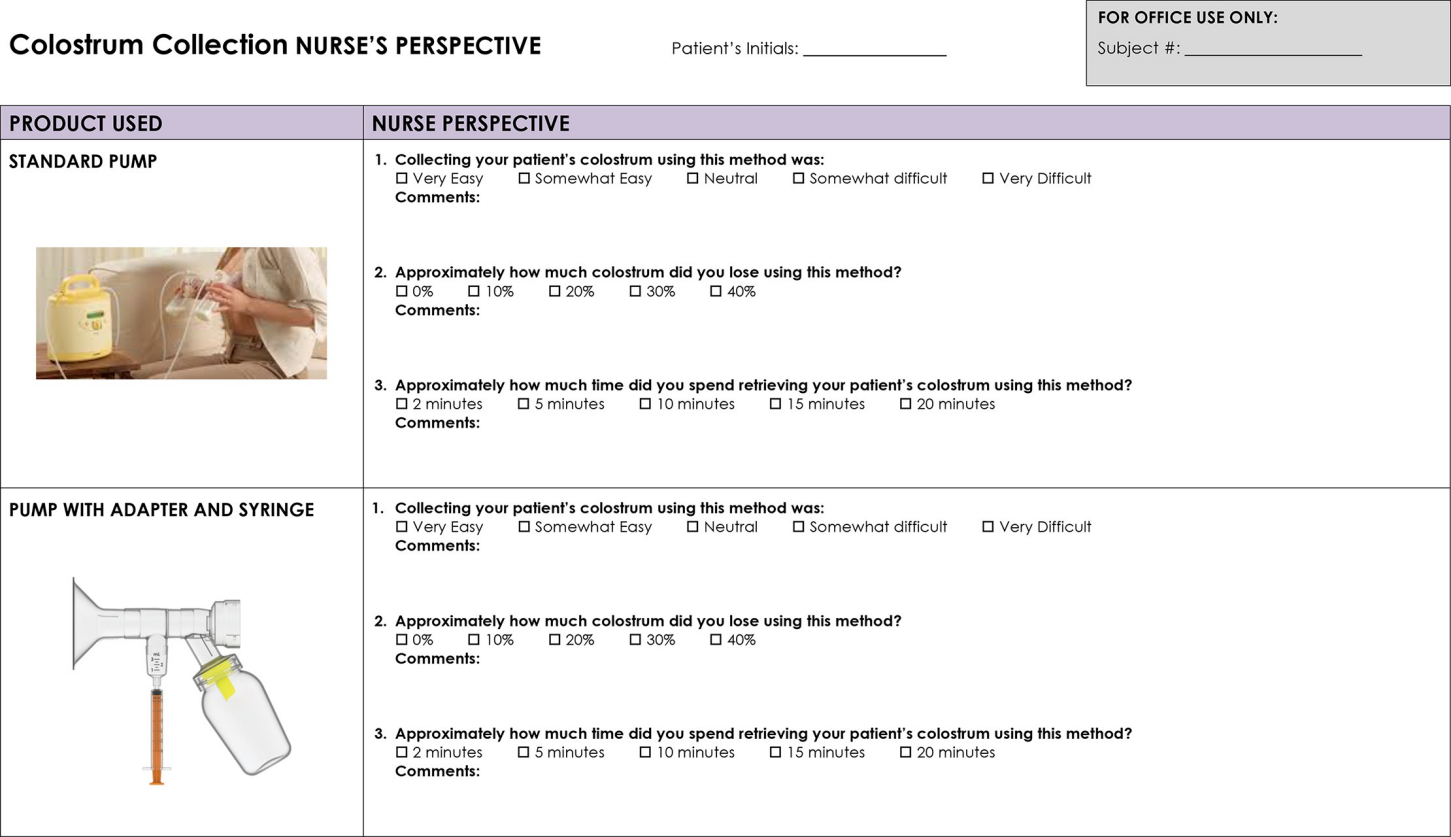
Nurse and lactation consultant survey.

**Fig 5:**
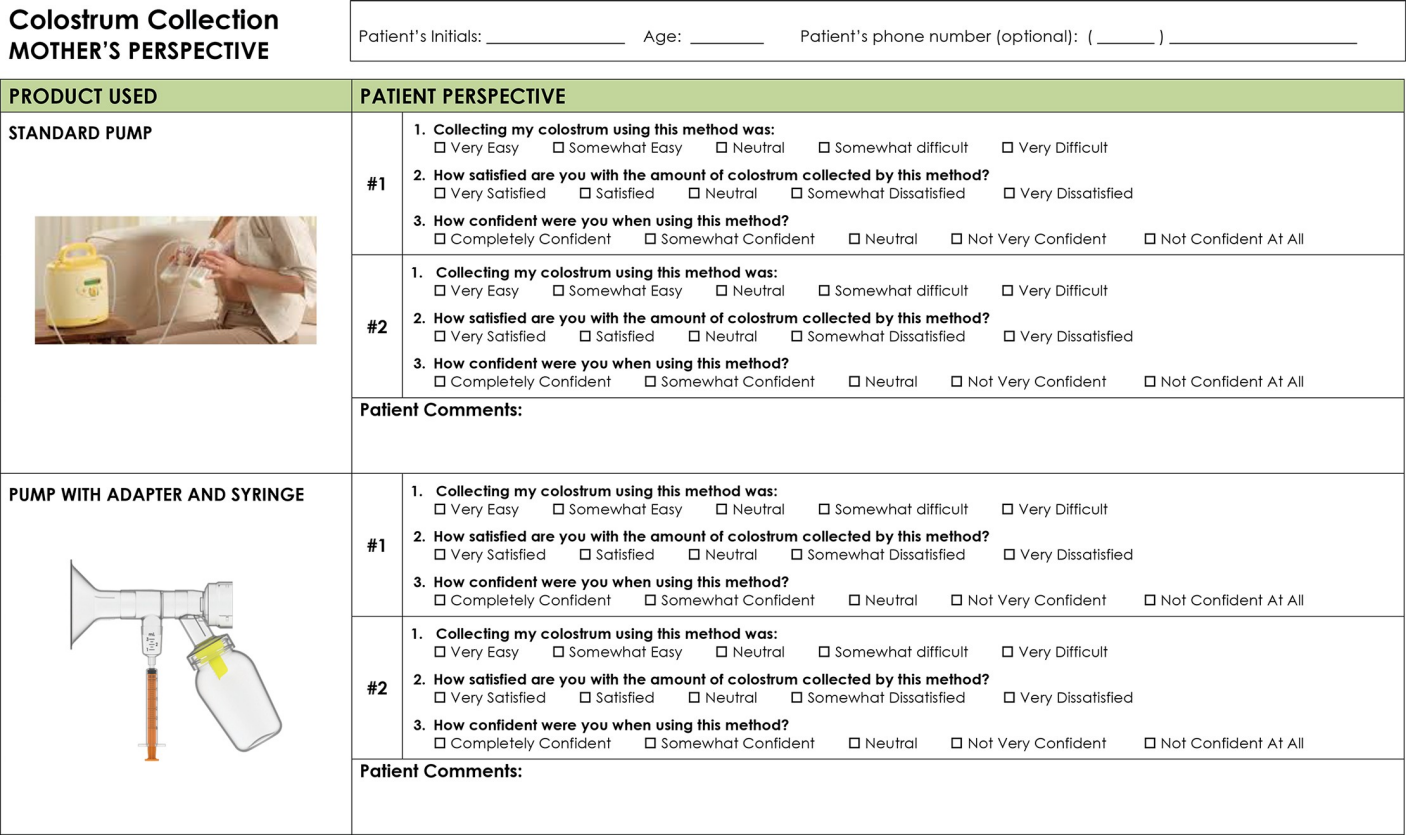
Patient survey.

An example of survey questions for both nurses or lactation consultants (Fig 4) and mothers (Fig 5) were completed at the conclusion of each mother’s colostral phase.

Data analysis: Data were analyzed with Stata (StataCorp, College Station, TX). Data were assessed using the Wilcoxon rank sum test. In addition to the quantitative analysis of the survey results, written open-ended responses of participants were analyzed using established grounded theory methods [14]. The constant comparison method was used to generate theory [15]. An independent data analyst conducted line-by-line coding, categorized codes and identified key themes.

## Results

### Quantitative results

Overall, 75 mothers were approached for the study, but eight were unable to collect colostrum. Therefore, 67 mothers were enrolled in the study. Demographic information is summarized in Table 1. Of 59 mothers with known parity, 61% (36/59) were primiparous and 39% (23/59) were multiparous.

**Table 1:** Demographics (n = 67).

	*Median*	*Interquartile range*	*Total range*
**Maternal age (years)**	29	21–40	17–43
**Gestational age (weeks)**	32	26–37	25–38
**Birthweight (grams)**	1535	800–2850	590–4450

Using a 5-point Likert scale to measure satisfaction, patients reported greater ease of use and increased confidence with the colostrum collection system for both hand and pump (p<0.01) compared to standard practice. Findings are summarized in Table 2.

**Table 2:** Patient satisfaction (n = 67).

	*Ease* *Mean (SD)*	*Median* *(IQR)*	*p*	*Satisfaction* *Mean* *(SD)*	*Median* *(IQR)*	*p*	*Confidence* *Mean* *(SD)*	*Median* *(IQR)*	*p*
**Hand only**	3.1 (1.2)	2 (3)	(ref.)	3.1 (1.2)	2 (3)	(ref.)	3.5 (1.1)	1 (4)	(ref.)
**Hand + PL**	3.9 (1.0)	2 (4)	< .01	3.8 (1.0)	2 (4)	< .01	4.1 (1.0)	1.5 (4)	< .01
**Pump** **only**	3.1 (1.4)	2.5 (3)	(ref.)	2.9 (1.3)	2 (3)	(ref.)	3.6 (1.2)	2 (4)	(ref.)
**Pump + PL**	4.1 (1.2)	1 (5)	< .01	3.8 (1.3)	2 (4)	< .01	4.3 (1.1)	1 (5)	< .01

SD = Standard Deviation, IQR = Interquartile range

Possible scores ranged from 1 to 5 with 5 being best (i.e. “Very Easy”, “Very Satisfied”, “Completely Confident”) and 1 being worst (i.e. “Very Difficult”, “Very Dissatisfied”, “Not Confident at All”).

Observations were collected from 89 nurses and lactation consultants, and findings are summarized in Table 3. There were more nurses and lactation consultants than patients because some patients had two nurses or lactation consultants who participated. Similarly, using a 5-point Likert scale to measure satisfaction, nurses and lactation consultants observed their patients had a better experience with the colostrum collection system for both hand and pump compared to standard practice. Nurses and lactation consultants reported less milk lost and shorter collection times with the use of the colostrum collection system. The collection times were not significantly different at the level of p ≤ .01.

**Table 3:** Nurse and lactation consultant observations (n = 89).

	*Ease* *Mean (SD)*	*Median* *(IQR)*	*p*	*Percent Loss* *Mean* *(SD)*	*Median* *(IQR)*	*p*	*Collection Time (minutes)* *Mean (SD)*	*Median* *(IQR)*	*p*
**Hand only**	3.1 (1.2)	3 (2)	(ref.)	13.0 (11.5)	10 (20)	(ref.)	6.6 (7.8)	5 (3)	(ref.)
**Hand** **+ PL**	4.2 (0.9)	4 (1)	< .01	7.6 (14.5)	0 (10)	< .01	6.2 (6.6)	5 (3)	0.35
**Pump** **only**	3.1 (1.4)	3 (2)	(ref.)	14.0 (15.7)	10 (20)	(ref.)	8.1 (5.3)	5 (10)	(ref.)
**Pump + PL**	4.1 (1.1)	4 (1)	< .01	9.4 (21.9)	0 (10)	< .01	7.5 (5.5)	5 (13)	0.03

SD = Standard Deviation, IQR = Interquartile Range

For “Ease,” the possible score ranged from 1 to 5 with 5 being “Very Easy” and 1 being “Very Difficult.”

For complete quantitative data please see S1 Dataset.

### Qualitative results for patients

Themes common for standard practice included difficulty with colostrum collection and wasted colostrum. Themes common for pump with adapter and syringe included greater colostrum collection and ease of use. Narrative data from mothers are shown in Table 4. Not all mothers provided qualitative comments. Upon review of the comments on all questionnaires, eight mothers reported that the slip-tip syringes fell off from the collection cup on the pump adapter or hand expression funnel. Scores for the device with a slip-tip syringe were higher in instances when the syringe stayed connected for the duration of the expression period. Two mothers commented that the pump with adapter and syringe had less suction strength than the standard pump. Eleven respondents reported that they appreciated the new system, for example: “Best way is to collect directly into a syringe,” and “I’m so happy because I collected colostrum for my baby with this system.”

**Table 4:** Patient perspectives of Primo-Lacto versus standard practice.

**Standard Pump**	
Collection difficult	*“Difficult collection due to how sticky the colostrum is*.*”*
Wasted colostrum	*“I found it was hard to get the full amount pumped into the container*. *It felt like I was wasting colostrum*.*”*
Couldn’t “get enough”	*“Still felt like couldn’t get enough drops to give to baby*.*”*
**Pump with Adapter and Syringe**	
Disconnected from syringe	*“Easy collection*, *but the cup connected to the syringe easily came disconnected*.*”*
Collected more colostrum	*“Using this new device I was able to collect small amount of colostrum that would have been lost using the regular pump equipment*. *Having a preemie in the NICU makes it that much more important to collect even small drops*!*”*
Ease of use	*“This made pumping way easier and faster*. *Collected twice as much*.*”* *“Pump with adapter and syringe was really easy and encouraging*. *Loved it*!*”*
**Standard Hand Expression**	
Collection difficult	*“Sticky colostrum difficult to get off of nipples and into bottle*.*”*
Wasted colostrum	*“With minimal amounts of colostrum coming out*, *expressing using this method made it impossible to catch/store all of my drops*.*”*
**Hand Expression with Funnel and Syringe**	
Syringe came off	*“Syringe slipped off*.*”*
Collected more colostrum	*“I felt like I was waiting much less*, *if any*.*”*
Ease of use	*“This method was much easier to collect/ store the colostrum*.*”*

### Qualitative results for clinicians

Themes found in standard practice include loss of colostrum. Themes found with the colostrum collection system include syringe coming off too easily and ease of use. Clinician narrative results are summarized in Table 5. Not all clinicians provided qualitative comments. Upon review of the comments on all questionnaires, we learned that in eight cases, no colostrum was being expressed with either standard practice or with the colostrum collection system. Clinicians reported five cases where the syringe popped of either the adapter of funnel. Lastly, we learned that in some cases the colostrum was so thick, that it was not draining fast enough down into the syringe from the hand-expression funnel.

**Table 5:** Nurse and lactation consultant perspectives on Primo-Lacto® versus standard practice.

**Standard Pump**	
Obstructed by valve	*“Caught in pump parts*.*”*
**Pump with Adapter and Syringe**	
Syringe came off	“*The one difficulty that the patients and I encountered was that the connection between the collection devices and syringe was too loose*, *causing the syringe to become inadvertently dislodged*, *resulting in wasting of the colostrum collected*. *This caused much distress*. *However*, *it is my understanding that this problem has been addressed*.*”*
Easier flow	*“Milk flowed easily into collecting chamber*.*”*
**Standard Hand Expression**	
Could not get into syringe	*“Couldn’t get drops off of side of bottle*.*”*
**Hand Expression with Funnel and Syringe**	
Syringe came off	“*There was a problem with the slip fit syringe falling off and a need to secure the syringe tighter*.*”*
Ease of Use	*“The equipment was simple to assemble and use*, *from my standpoint*, *and also according to feedback received from moms*. *Several features made colostrum collection much easier*, *more efficient*, *and with little wasting of colostrum*. *The “V” shaped collection funnel*, *manufactured with materials which reduced friction really helped in the collection of the initial thick viscosity drops*.*”*

## Discussion

This system assists in colostrum collection and delivery in circumstances where direct breastfeeding is impossible or undesired. The colostrum collection system is designed to *capture* more colostrum with greater ease for both the mom and clinician, and the results prove this goal was achieved. The colostrum collection system does not help a mother increase her supply of colostrum. If mothers want to produce more, they need to follow a lactation consultant’s recommendations.

The colostrum collection system addresses issues of uncertainty or variations in patient care by providing a dedicated device to help set realistic expectations for a new mother and allow clinicians an efficient, clean way to transport, store and administer the mother’s colostrum to the neonate. Any new mother is exhausted, and a mother who has just given birth to an infant who is preterm or who has other health issues has added stress. A better experience with expression and pumping in the hospital may lead to a higher incidence of mothers who continue to pump milk and/or breastfeed their child upon discharge. As we see in Table 4, the notion of not being able to “get enough” may lead to feelings of “I’m not enough,” inadequacy or failure thereby threatening successful lactation [16]. If hospitals adopt a clinically proven way to manage colostrum collection, storage, and application, hundreds of thousands of babies may have an improved chance to obtain the biological nutrition their bodies are designed to receive. In addition, mothers will be given the best opportunity to initiate and continue successful breastfeeding.

NICU patients are about 0.15 percent of the population in the United States, yet they lead to 0.45 percent of overall healthcare expenses. On average, it costs $3,000 per day to hospitalize an infant in the NICU. The average employer cost of a full-term infant is $2,830, compared to $41, 610 for a preterm infant. For extremely preterm infants, the cost can easily exceed $250,000 [17]. Studies show that infants who received oral colostrum priming, relative to those who did not, reduced the median length of stay by 16 days [1]. That is a $48K savings for each infant—a mother’s colostrum is quite literally “liquid gold”. This savings is particularly relevant to insurance companies and employers.

Conducting a study helps us to understand a product’s shortcomings, making it clear what needs to be changed in the design for clinical success. In response to the qualitative comments, we improved the hand expression funnel by making it shorter, facilitating faster travel of colostrum to the syringe, and changing the pump adapter design to accommodate an ENFit syringe. Because ENFit syringes have a thread incorporated into their design, the syringe screws into the base of the colostrum collection system collection cup, making it impossible to disconnect until desired. Additionally, the collection cup itself now screws onto the pump adapter, making it impossible to disconnect. To address vacuum loss, the length of the adapter’s shaft has been shortened and the hole inside the adapter’s blocking wall has been adjusted to maximize vacuum.

Limitations include the size of the study (67 mothers, 89 nurses or lactation consultants) and that all patients enrolled spoke English due to costs to provide translated surveys and assign multi-lingual nurses or lactation consultants to the study. In addition, at the time of the study, the colostrum collection system could only accommodate slip tip feeding syringes, as the molds for the ENFit syringe had not been produced yet. Each subject served as her own control. The sequences of events was not randomized and should be randomized in future studies as milk production increases over time. Actual volumes of colostrum were not measured with an instrument but were estimated by nurses or lactation consultants.

Further research should utilize standardized measurement tools to more precisely quantify volume of colostrum lost. Future research could also examine the efficacy of incentives for women to express colostrum, how the colostrum collection system affects post-discharge breastfeeding numbers, and health outcomes for babies receiving more colostrum due to the colostrum collection system.

Another area of research we are interested in exploring examines how this colostrum collection system may reduce anxiety among mothers unable to breastfeed after birth, thereby improving milk production as well as morale. More research with this colostrum collection system is needed to understand its’ impact on time saved for nurse or lactation consultant staff, health outcomes for babies receiving more colostrum due to the colostrum collection system, and overall healthcare system savings due to the colostrum collection system.

Primo-Lacto is a novel tool to help facilitate successful colostrum collection and improve the experience both for clinicians and patients. Not only does this device help mothers maximize colostrum captured, it also improves nurse and lactation consultant workflow by eliminating the need to transfer the colostrum into another delivery device. Removing this step may save time and reduce frustration. Lastly, research shows that feeding babies’ colostrum reduces health care costs.

## Supporting information

S1 Dataset Primo lacto data analysis. (XLSX)
